# Endoscopic transnasal approach to sellar tumors

**DOI:** 10.1016/S1808-8694(15)30098-7

**Published:** 2015-10-19

**Authors:** Rodrigo de Paula Santos, Samuel Tau Zymberg, Júlio Zaki Abucham Filho, Luis Carlos Gregório, Luc Louis Maurice Weckx

**Affiliations:** a Master’s and doctoral degrees, clinical head of the rhinology unit, Sao Paulo Federal University - Paulista School of Medicine.; b Doctor, assistant in the neurosurgery discipline, Unifesp/EPM.; c Doctor, professor of endocrinology, Unifesp/EPM, Chefe do setor de Neuroendocrinologia da Unifesp/EPM.; d Doctor, professor of the ENT and head & neck department, Unifesp/EPM, head of the otorhinolaryngology discipline, Unifesp/EPM.; e Full professor, head of the ENT and head & neck department, Unifesp/EPM. Paulista School of Medicine (EPM) / Unifesp.

**Keywords:** endonasal, endoscopic, pituitary, minimally invasive, transsphenoidal

## Abstract

Transsphenoidal surgery for sellar region tumors is traditionally done only by neurosurgeons. The use of endoscopes has permitted a direct transnasal approach to the sphenoidal sinus, without dissection of the septal mucosa, reducing postoperative morbidity.

**Aim:**

The purpose of this study was to assess the technical difficulties, and per and postoperative complications of the otolaryngological management of the endoscopic transnasal approach to the sellar region.

**Material and Method:**

159 patients undergoing sellar region surgery between March 2001 and December 2006 were assessed retrospectively. 91 patients who underwent 95 endoscopic transnasal procedures were included in this study. Study design: a clinical retrospective study.

**Results:**

The endoscopic transnasal technique was feasible for every patient, independent of age, anatomical variations, tumor characteristics, tumor etiology, and previous surgical history. There was no need to remove the middle turbinate or septal deviations in any of the cases. The most significant peroperative complication was CSF leak during tumor removal (13.68%). Postoperative complications were: nasal bleeding (8.42%), CSF leak (8.42%), and meningitis (2.19).

**Conclusion:**

The transnasal endoscopic approach was accomplished with minimal invasion, preserving nasal structures in all 95 procedures, independent of age, anatomical variations, tumor characteristics, tumor etiology, and previous surgical history..

## INTRODUCTION

Sellar tumor surgery is traditionally performed by neurosurgeons. Otorhinolaryngologists, however, have become important partners in the surgical treatment of pituitary adenoma patients since the return of the transsphenoidal approach for sella turcica access in the 1960s. The transsphenoidal approach, which traditionally was done by neurosurgeons, has received contributions based on the knowledge that otorhinolaryngologists have about nasosinusal surgery, which has supported sella turcica exposure and minimized injury to nasal structures. In many medical centers neurosurgeons and otorhinolaryngologists are part of a surgical team for hypophyseal surgery, which reduces complication rates such as septal perforation, cerebrospinal leaks and functional nasal problems.

The Italian surgeon Davide Giordano idealized the transsphenoidal approach in 1897 based on his anatomical study of cadavers. Giordano proposed a transglabellartransfacial approach to the sella turcica. Herman Schloffer,^1^ a Viennese surgeon, successfully put in practice this approach for the first time in 1907.

Oskar Hirsch, also from Vienna, was the first surgeon to practice the endonasal transsphenoidal approach, avoiding a lateral rhinotomy.^2^ Albert Halstead, from Chicago, introduced the sublabial approach as a variant; Harvey Cushing popularized this approach and performed over 200 such procedures for removing pituitary tumors.^3^ The frontal craniotomy approach eventually substituted this technique, given limitations such as a narrow operating field, poor illumination and the risk of infection.

In South America, the Argentinean otorhinolaryngologist Eliseo Victor Segura (1870-1946) - never cited in more recent medical papers - modified and improved the endonasal technique that Hirsch had described in 1910. Furthermore, Segura personally designed all of the surgical tools he deemed necessary for this procedure; his brother produced these instruments.^4^

Interest in the transsphenoidal approach to the hypophysis was reawakened and disseminated worldwide only in the 1960s. The factors that led to this development were the introduction of image intensifiers (scopes) to confirm the surgical approach, by Gerard Guiot5 from France, and of surgical microscopes that provided superior lighting and magnification, by Jules Hardy6 from Montreal. Since then the transseptal-transsphenoidal technique has been the standard approach to pituitary surgery and sellar tumor resection.

The increased popularity of nasosinusal endoscopic surgery in otorhinolaryngology created a new area of interest, namely its applicability to pituitary surgery. Its use enabled direct transnasal access to the sphenoidal sinus without the need to detach the nasal septum, which reduced postoperative discomfort and morbidity compared to traditional methods.^7^, ^8^, Guiot et al.^9^ in the 1960s recognized the utility of endoscopes in pituitary surgery, based on his endoscopic investigation of the sellar content during classical transsphenoidal approaches, which made it possible to expand the visual field to previously inaccessible regions. In 1992 Jankowski et al.^10^ operated three patients that had pituitary adenomas, using an endoscope for a direct transnasal approach without using a surgical microscope. Sethi and Pillay^11^ used nasal specula in a transseptal endoscopic approach. Jho and Carrau^12^ systematized the direct endoscopic approach to the sphenoidal sinus without the need to involve the nasal septum or other paranasal sinuses.

Since then, various technical variants for the endoscopic transnasal approach have been proposed, aiming to reduce surgical invasiveness. There are published papers from groups that perform surgery through one or both nostrils; that use a fixator to hold the endoscope for a neurosurgeon who operates without the participation of an otorhinolaryngologist; that use flexible endoscopes; that use nasal specula or not; that use nasal pads or not at the end of the procedure; and that remove the middle and/or superior turbinates or septal deviation to facilitate surgical access.

The aim of this paper was to verify the technical difficulties, intercurrences and postoperative complications of the otorhinolaryngological management of the endoscopic transnasal approach to the sella turcica.

## MATERIAL AND METHOD

After the Research Ethics Committee approved the study (CEP 1787/05), an analysis was made of the charts and images of patients that had undergone surgery for the removal of sellar tumors between March 2001 and December 2005.

All of the patients that underwent transnasal endoscopic surgery for the removal of sellar tumors, without the use of surgical microscopes, were included.

### Indication for surgery

Surgery was indicated for all the patients based on discussions about their clinical picture and radiological exams during the weekly case-discussion multidisciplinary meetings of the neuroendocrinology unit. Participants include endocrinologists, members of the image diagnosis unit, neurosurgeons and otorhinolaryngologists.

### Surgical technique

Placement of the patient and the team

**Figure 1:**
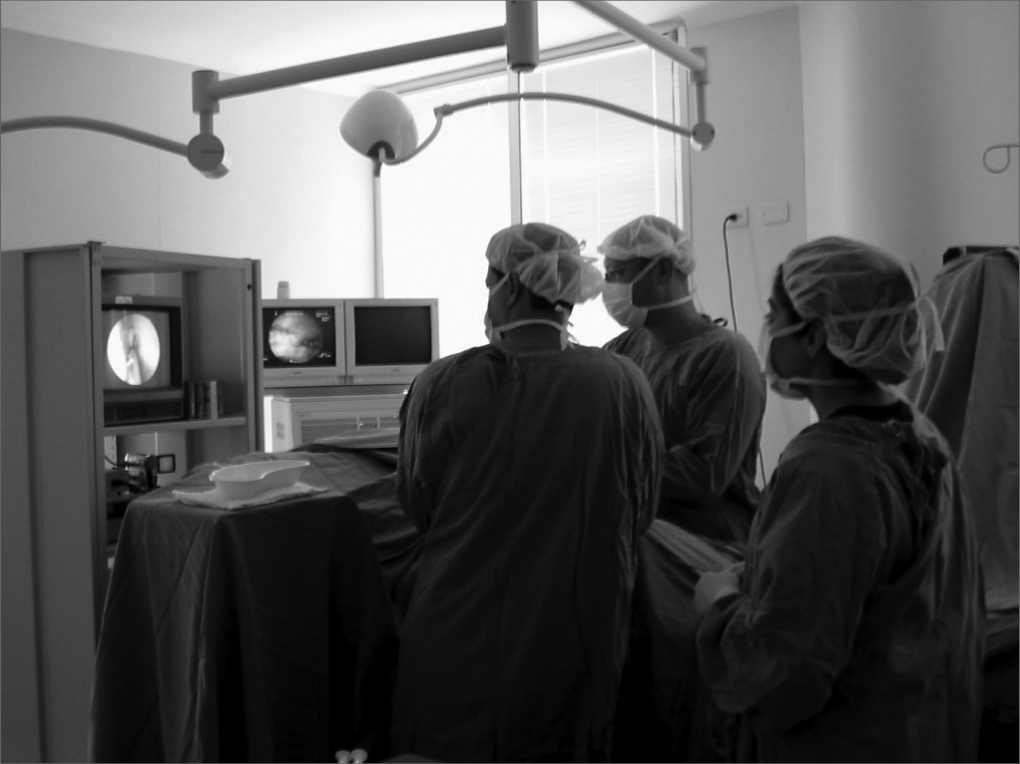
Arrangement of the surgical team.

Patients were placed supine on the operating table, under general anesthesia and orotracheal intubation. The back was elevated by about 10º, and the head was tilted 10º to the right. Vertical tilting of the head varied according to the site of the lesion. The head was slightly flexed for tumors that preferentially involved the sphenoidal sinus and the clivus; the head was placed in a neutral position or slightly hyperextended for tumors involving the suprasellar region and the sphenoidal plane. The otorhinolaryngologist was to the right of the patient and the neurosurgeon was to the left. The assistant and instrumentator was also to the right of the patient, next to the lower limbs. The anesthesiologist was to the left and next to the patient’s feet. The videoendoscopic surgery equipment (monitor, camera, light source and documentation equipment) was placed behind the patient’s head in such a way that both the neurosurgeon and the otorhinolaryngologist could comfortably look at the monitor. Aqueous chlorhexidine gluconate at 0.2% was used for antisepsis of the face and abdomen, after which sterile drapes were placed (Figure 1).

### Equipment

Rigid, 4mm diameter, 18 cm length, 0° and 45° angle endoscopes (Karl Storz, GmbH and Co, Tuttlingen, Germany). Images were recorded on VHS or mini-DV (digital) tapes.

### Identification of reference points in the nose

**Figure 2:**
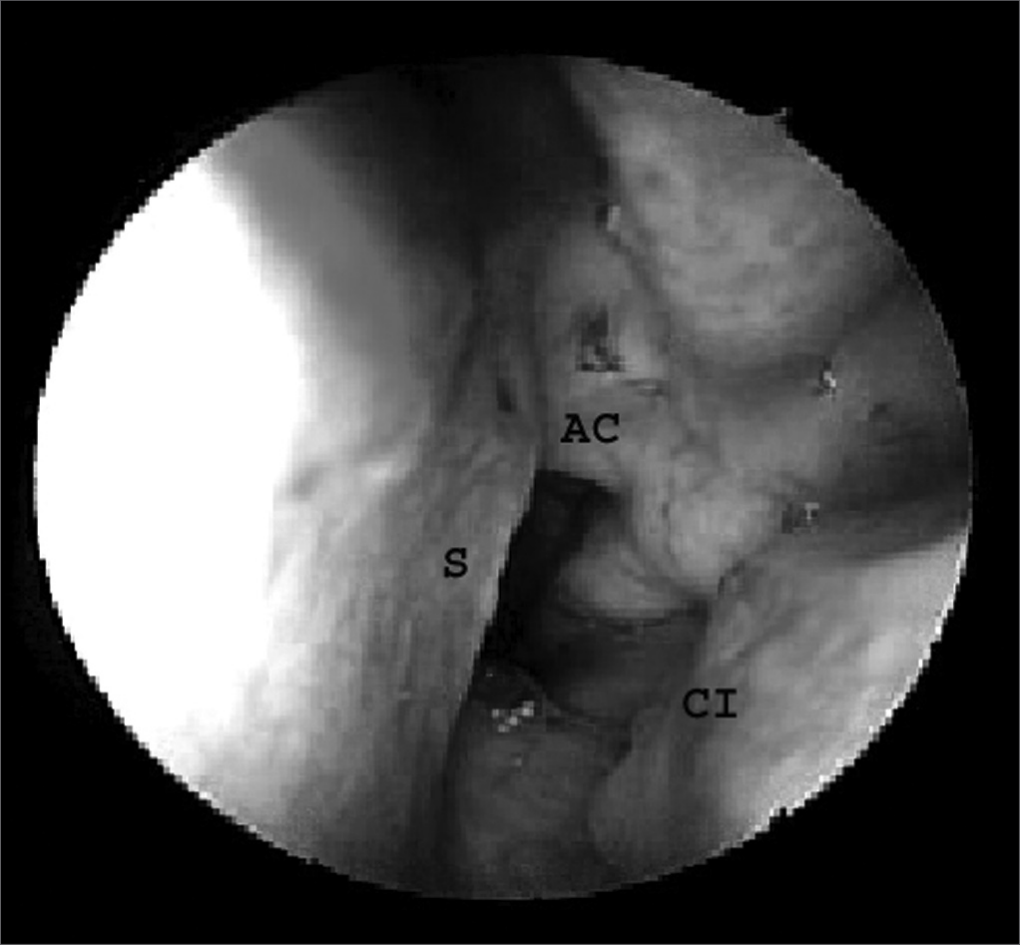
Nasal cavity landmarks. AC - Choanal arch, S - Nasal septum, CI - Lower turbinate.

The endoscope was introduced into one of the nostrils in parallel to the floor of the nasal cavity; the first structures to be identified were the head of the lower turbinate laterally and the nasal septum medially. The insertion of the middle turbinate was located superior and posterior to the lower turbinate. The endoscope was advanced along the nasal floor to the choana to identify its medial border, the vomer (nasal septum), its roof, the lower wall of the sphenoidal sinus (choanal arch) and its lateral aspect, the tail of the lower turbinate (Figure 2).

### Preparation of the nasal fossae

Under endoscopic view, long cottonoids imbibed in adrenalin at a 1:1000 concentration were placed in both nasal fossae between the middle turbinate and the nasal septum for vasoconstriction and decreased perioperative bleeding, and a cleaner surgical field. The cottonoids were removed after about five minutes and the middle turbinate was gently displaced laterally, avoiding fractures close to its insertion. The endoscope was then advanced to the sphenoethmoidal recess to locate the superior turbinate and the anterior wall of the sphenoidal sinus. Small cottonoids were placed in this region for two to three minutes. The space between the middle turbinate and the nasal septum was increased after removing these cottonoids, facilitating identification of the sphenoidal sinus ostium.

### Ostium of the sphenoidal sinus

**Figure 3:**
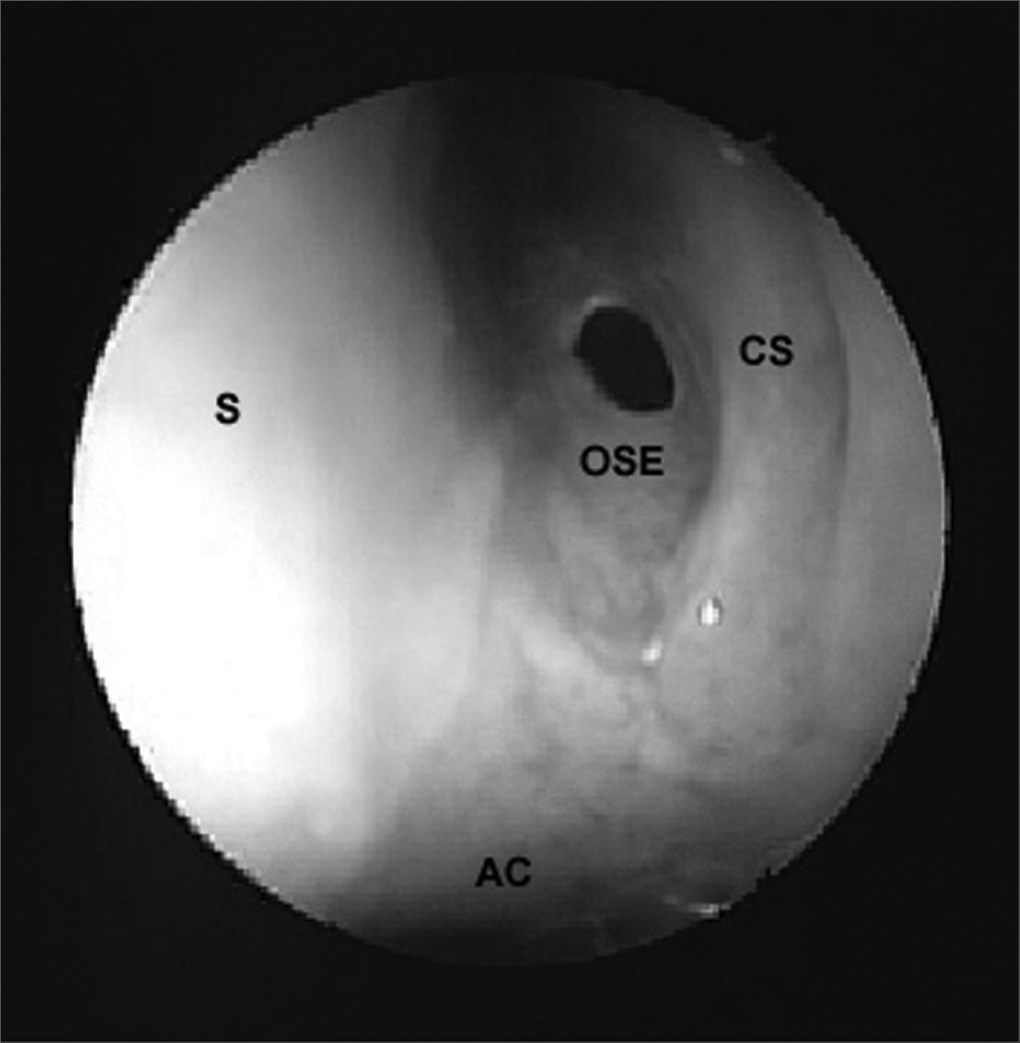
Ostium of the left sphenoidal sinus. AC - Choanal arch, S - Nasal septum, CS - Superior turbinate, OSE - Ostium of the sphenoidsl sinus.

Effectively, the first step of this surgery consisted of opening the sphenoidal sinus ostium. The most important landmarks to locate the sphenoidal sinus ostium are the choanal arch and the tail of the superior turbinate. The sphenoidal sinus ostium was located close to the tail of the superior turbinate, along the sphenoethmoidal recess, about 1.5cm above the choanal arch (Figure 3). When the ostium was not visible, the tail of the superior turbinate and occasionally that of the supreme turbinate were displaced laterally, after which the anterior wall of the sphenoidal sinus was gently palpated to find the point of lower resistance, which is the ostium, at times covered by redundant mucosa. A delicate Kerrison-type forceps or specific Stammberger sphenoidal sinus forceps was used to open the sphenoidal sinus ostium, moving initially downwards and medially to avoid injury to important anatomical structures placed superior and lateral to the sinus, such as the optic nerve and the internal carotid artery. The next step was wide removal of the anterior wall of the sphenoidal sinus. A similar procedure was done through the contralateral nasal fossa to attain a bilateral ample sphenoidotomy.

### Nasal and intersinus septa

**Figure 4:**
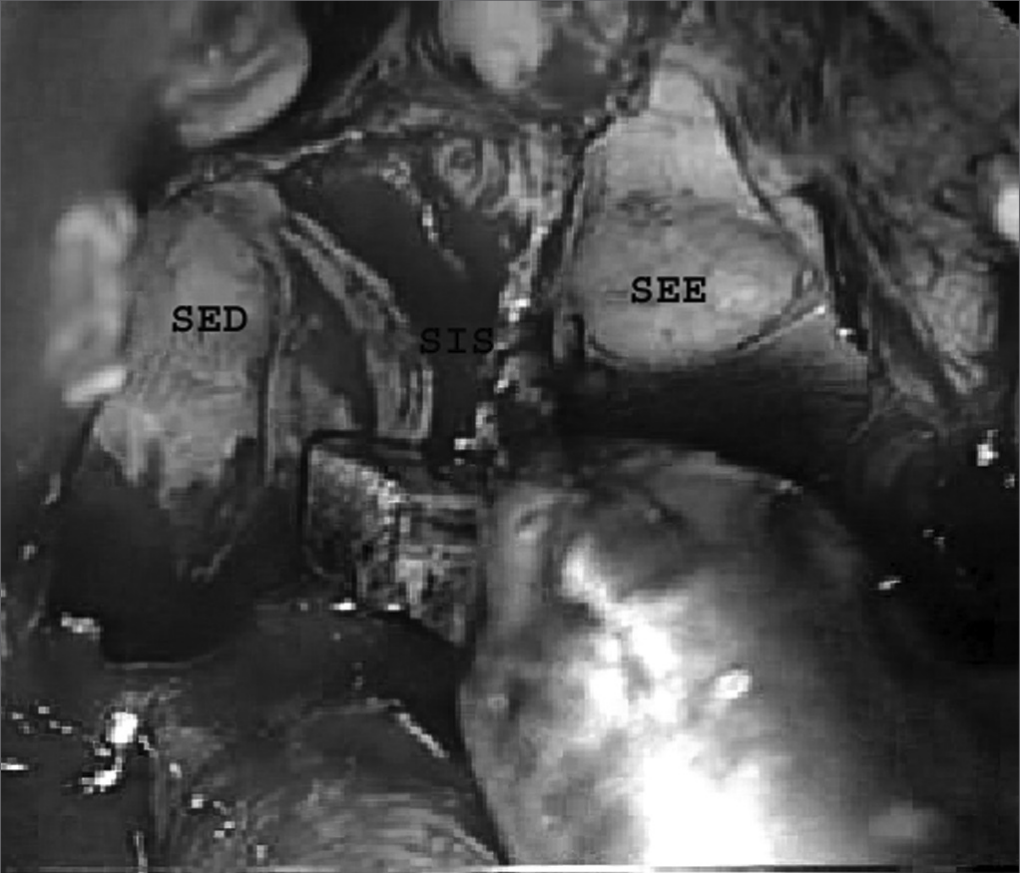
Removal of the intersinus septum. SES - Left sphenoidal sinus. SED - Right sphenoidal sinus. SIS - Intersinus septum.

About 1.0 to 1.5 cm of the posterior nasal septum was removed for simultaneous access to the sphenoidal sinuses through both nasal fossae. A Kerrison or a reverse cutting forceps was used to this end. The intersinus septum was carefully removed with cutting forceps to avoid accidental fractures of the sellar floor. A simultaneous approach through both nasal fossae was used from this point onwards; the otorhinolaryngologist was positioned next to the right nasal fossa and the neurosurgeon was positioned to the left nasal fossa. There may be other always incomplete vertical or oblique septa in the sphenoidal sinus, other than the intersinus, sagittal and generally paramedian bone septum that completely separates the right and left sphenoidal sinuses. There septa were removed with cutting forceps only when it became necessary to improve the access to the sellar region, and only after a careful analysis of image exams (computed tomography and/or magnetic resonance imaging) that revealed the anatomical relations between these septa and adjacent structures (Figure 4).

At this point the complete anatomy of the sphenoidal sinus and its main landmarks were identified. This step is extremely important for correct orientation when opening the floor of the sella.

### Identifying landmarks in the sphenoidal sinus

**Figure 5:**
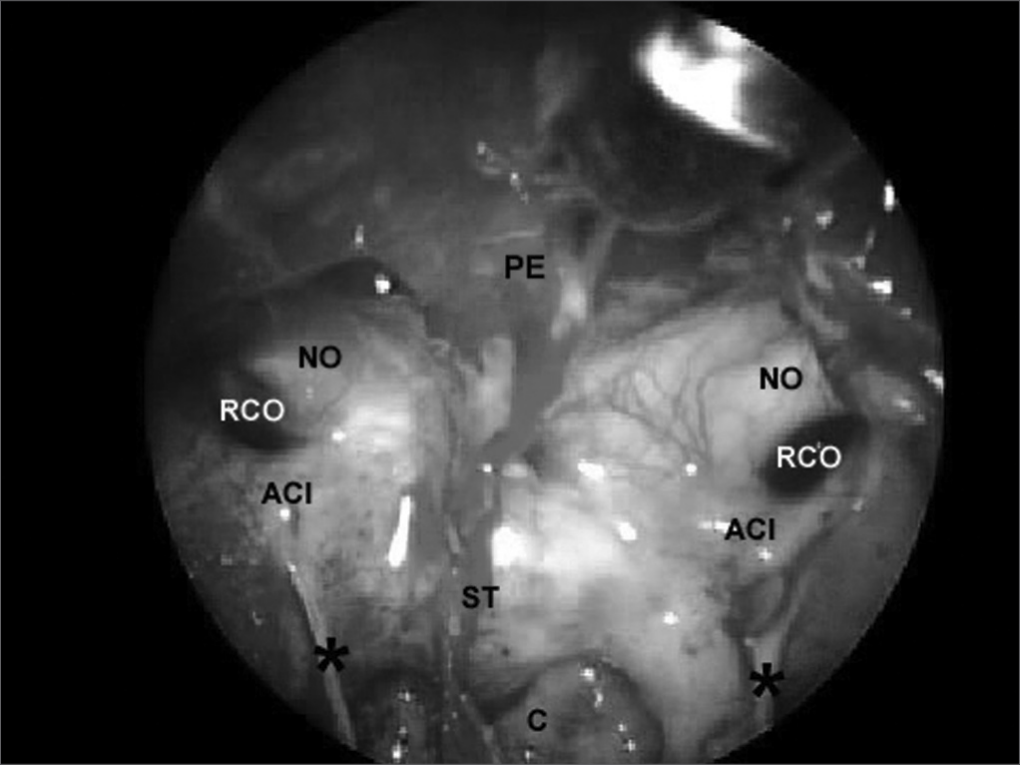
Sphenoidal sinus after partial removal of the intersinus septum. PE - Sphenoidal plane. NO - Optic nerve, ACI - Internal carotid artery, RCO - Carotid-optic recess, ST - Sella turcica, C - clivus, * - incomplete septa within the sphenoidal sinus.

After removal of the intersinus septum, the sphenoidal sinus could be compared to a pyramid with its base pointing anteriorly. Figure 5 shows:

Posterior wall (smaller base of the pyramid), where the floor of the sella forms its upper part and the clivus forms the lower part; it is defined laterally by the carotid prominences, superiorly by the sphenoidal plane and inferiorly by the floor of the sphenoidal sinus;

Lateral walls, delimited superiorly by the optic nerve prominence, inferiorly by the bone prominence that lines the second trigeminal nerve branch, posteriorly by the carotid prominence and anteriorly by the anterior wall of the sphenoidal sinus;

The roof, formed by the sphenoidal plane, delimited posteriorly by the floor of the sella, anteriorly by the anterior wall of the sphenoidal sinus and laterally by the optic nerve prominences;

The floor, where the clivus forms the posterior portion and the sphenoidal rostrum forms the anterior portion. The extension by which the clivus participates in forming the posterior and inferior walls of the sphenoidal cavity varies according to the degree of sinus pneumatization. The floor is continuous with the walls laterally, and with the sphenoidal sinus wall anteriorly.

The floor of the sella turcica may be located below the sphenoidal plane, above the clivus, between the carotid prominences.

Sphenoidal sinus pneumatization was proportional to the number of anatomical landmarks. Sphenoidal pneumatization may be classified into three types: the conchal type, which does not reach the sphenoidal body, is a small sinus that is separated from the sella turcica by a thick bony wall; the pre-sellar type, in which the posterior border of the sinus does not go beyond the lower half of the sellar floor; and the sellar type - the most frequent type - in which the cavity extends below the sella until reaching the clivus.^13^ Generally not all of these landmarks are located; locating the sphenoidal plane, the clivus and the bone prominences of the internal carotid arteries is sufficient to safely establish the borders of the sellar floor. The image intensifier may be used in cases of pre-sellar or conchal sinuses and insufficient anatomical landmarks to confirm the surgical trajectory.

From this point onwards the procedure is done collectively; the otorhinolaryngologist handles the endoscope and the aspirator through the right nasal fossa and the neurologist handles the instruments through the left nasal fossa. The following steps are: opening the sellar floor, opening the dura-mater, removing the tumor, exploring the sella and reconstructing the sella (if there is a cerebrospinal leak).

### Exploring the sella

**Figure 6:**
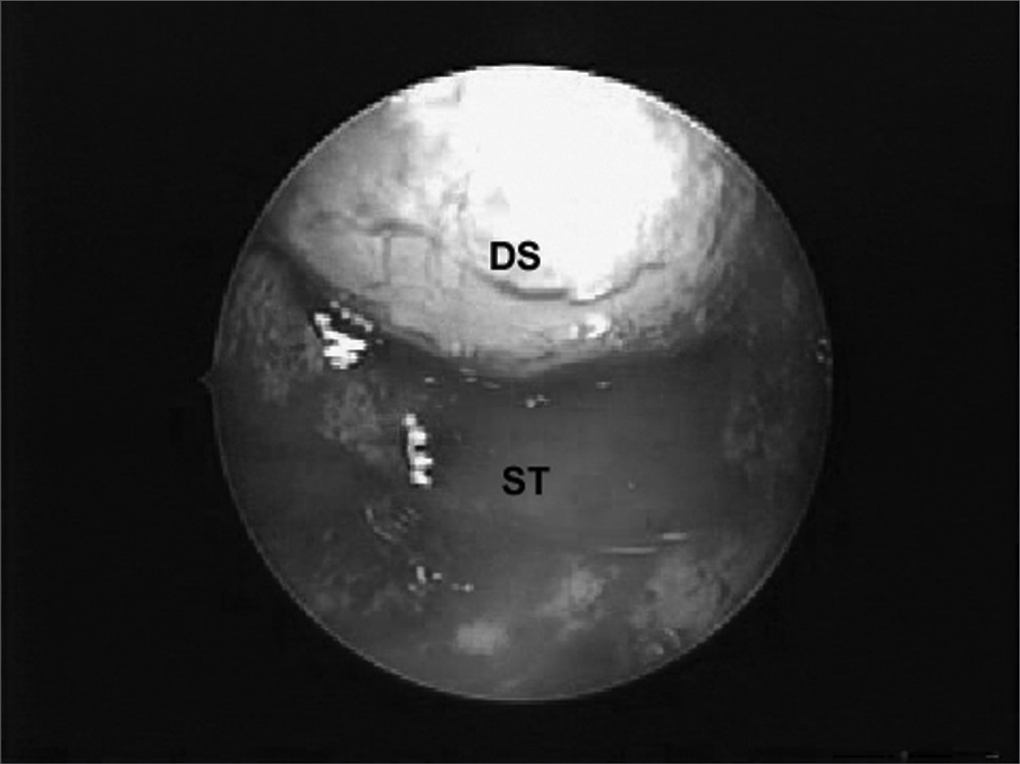
Sellar diaphragm, inverted within the sella, after removal of a macroadenoma. DS - Sellar diaphragm, ST - Sella turcica.

Careful exploration of the sella with the 45º endoscope to find tumor remains and holes in the sellar diaphragm that could result in cerebrospinal leaks is done after removal of the tumor (Figure 6).

### Reconstructing the sella

Tamponade of the sella with Surgicel® and fat harvested from the periumbilical region and fixated with biological glue (Beriplast®) is done if there is a cerebrospinal leak after the procedure. There was no need for sellar reconstruction if the sellar diaphragm was intact. Lumbar tubes were not routinely used in cases of cerebrospinal leaks.

Patients were extubated in the operating room at the end of the procedure and sent to the postanesthetic care unit; after recovery, patients were sent to the endocrinology unit wards. There was no need for intensive care units.

### Assessment criteria

The following items were assessed: the feasibility of this technique for all cases of sellar tumors, taking into account the patient’s age; anatomical variants; tumor characteristics and etiology; the need for removing the middle turbinate and for correcting septal deviation; the need for using the image intensifier (scopy) during surgical access to confirm the trajectory of instruments; the occurrence of nasal bleeding and the need for procedures (such as nasal tamponade and cauterization) to control bleeding; the occurrence of cerebrospinal leaks during and after surgery and the need for procedures for correcting those leaks; and the occurrence of postoperative meningitis.

## RESULTS

The files of 159 patients that underwent sellar region surgery between Marcy 2001 and December 2005 were analyzed. This study included 91 patients that had undergone 95 endoscopic transnasal procedures. The remaining 68 patients, in whom surgery was done using the sublabial approach with the microscope and endoscopic review of the sellar region, were excluded. Of 91 patients, 35 were male and 56 were female; the mean age was 47.6 years, extending from 9 to 79 years.

Transnasal endoscopic sellar region surgery was done in patients bearing the following diagnoses: secreting and non-secreting pituitary adenomas, craniopharyngiomas, cordomas, Ratke’s pouch cysts, metastases from other sellar tumors and lymphocytic hypophysitis.

Out of 91 patients, four underwent a second transnasal endoscopic procedure; three of them had non-secreting adenomas and one patient had a sellar metastasis originating from a breast adenocarcinoma. (Table 1).

**Table 1:** Etiological diagnosis and number of procedures (N (%)).

Diagnosis	Patients	Surgery
Non-secreting pituitary adenoma	46 (50,5)	49 (51,5)
Secreting pituitary adenoma	36 (39,6)	36 (37,9)
Craniopharyngioma	4 (4,4)	4 (4,2)
Cordoma	1(1,1)	1 (1,1)
Ratke’s pouch cyst	2 (2,2)	2 (2,1)
Metastases from other sellar region tumors	1 (1,1)	2 (2,1)
Lymphocytic hypophysitis	1 (1,1)	1 (1,1)

Total	91 (100,0)	95 (100,0)

Eighteen patients had already been operated by an alternative surgical access route, as follow: craniotomy - five patients, and the sublabial approach - 13 patients. Of 13 patients operated sublabially, two had undergone two surgical interventions and one had undergone three interventions.

**Figure 7:**
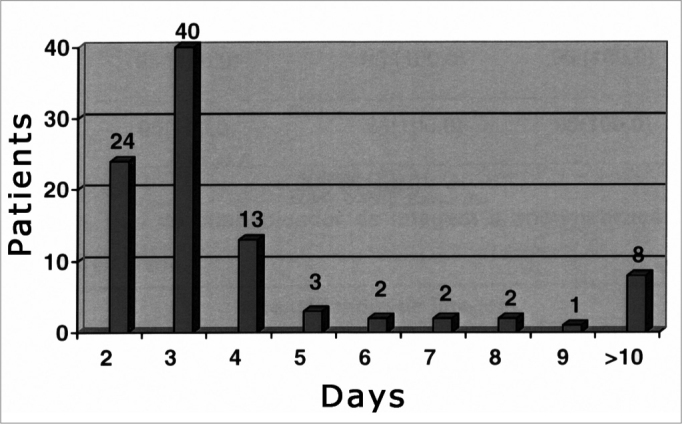
Distribution of patients according to the hospital stay.

Patients remained in the hospital for up to four days in 81.05% of the surgical procedures; patients were in hospital for over ten days in 8.42% of the procedures (Figure 7).

The transnasal endoscopic technique was done in all of the sample regardless of age, anatomical variants, tumor characteristics and etiology and previous surgery. There was no need to remove the middle turbinate or to correct septal deviation for surgical access in any of the cases. The image intensifier was used to confirm the trajectory of surgical instruments in two cases that had a conchal type sphenoidal sinus (Table 2).

**Table 2:** Technical difficulties (N (%)).

	Removal of the middle turbinate	Removal of septal deviation	Image intensifier	Total
Yes	0 (0,0)	0 (0,0)	2 (2,1)	2 (2,1)
No	95 (100,0)	95 (100,0)	93 (97,9)	93 (97,9)

Total	95 (100,0)	95 (100,0)	95 (100,0)	95 (100,0)

There was no perioperative bleeding that required the surgical procedure to be interrupted. No patients required blood transfusion.

The main intercurrence was opening of the sellar diaphragm during tumor removal, which led to cerebrospinal fluid leakage and the need to fill in the sella with fat and biological glue (Beriplast®). Sellar diaphragm periopeartive injury was repaired in 13 cases (13.68%); external lumbar drainage was done in two of these cases (3.15%) as adjunct treatment (Table 3).

**Table 3:** Intercurrences (N (%)).

	Nasal bleeding	Injury to the sellar diaphragm	Total
Yes	0 (0,0)	13(13,7)	13(13,7)
No	95 (100,0)	82(86,3)	82(86,3)

Total	95(100,0)	95(100,0)	95(100,0)

Eight patients (8.42%) had postoperative nasal bleeding that required otorhinolaryngological care. Nasal bleeding occurred during the immediate postoperative period in six of these cases, on the fifth day in one case and seven days after surgery in one case. One of the cases was solved with lavage of the nasal cavity with saline. Bleeding was controlled by placing Gelfoam® within the nasal fossae for 24 hours in one patient. Anterior nasal tamponade was done in three cases; tamponade was kept in place for 24 hours in two cases and for 48 hours in one case. Two patients required nasal anterior and posterior tamponade, which was kept in place for 24 hours in one patient and for 72 hours in the other. One patient required surgical cauterization of the nasal mucosa.

Eight patients (8.42%) developed cerebrospinal leaks postoperatively; two of them were identified on the first postoperative day, two on the third postoperative day, two on the fourth postoperative day and two on the thirds week after surgery. Of the six patients in which the leak was detected during the first week postoperatively one was kept under medical observation until spontaneous resolution of the leak. External lumbar drainage was done in two cases. Endoscopic surgery was done in one patient; this procedure was associated with external lumbar drainage in two patients. Endoscopic surgery was done in the two patients that developed leaks in the third week postoperatively. Bacterial meningitis was a complication in two cases (2.19%); both developed cerebrospinal leaks that were identified in the third week postoperatively (Table 4).

**Table 4:** Complications (N (%)).

	Nasal bleeding	Cerebrospinal leak	Meningitis	Total
Yes	8 (8,4)	8 (8,4)	2 (2,1)	18 (18,9)
No	87 (91,6)	87 (91,6)	93 (97,9)	77 (81,1)

Total	95 (100,0)	95 (100,0)	95 (100,0)	95 (100,0)

## DISCUSSION

Transnasal endoscopic surgery of sellar region tumors is part of the current search for the so-called minimally invasive procedures. We can see a transition from craniotomy to a transsphenoidal approach, initially done externally and then endonasally.^2^, ^4^

Guiot et al.^9^ introduced endoscopes into sellar region surgery as an accessory tool for the microscope to improve visualization. Jankowski et al.^10^ later used it in place of the microscope as the only tool for visualization of the sphenoidal sinus directly in a transnasal approach. Jho and Carrau^12^ systematized this approach and used it in 48 procedures with no need to detach the nasal septum, which meant that the access route became less invasive.

As of March 2001 at our institution, otorhinolaryngologists decided to take part of sellar region transsphenoidal surgery, performing an endoscopic review after tumor resection, which at the time was done with a microscope through a sublabial approach. Since 2003 the procedures started to be done using an endoscopic transnasal approach without the microscope. Other authors have also reported a transition period between the microscope and the endoscope techniques; this allows the neurosurgeon to gradually adapt to the endoscope while retaining the possibility of using the microscope at any moment if judged necessary.^12^, ^14^

There are various surgical technical options for accessing the sellar region when the endoscope becomes the only visualization tool. Endoscopic access may be transeptal, transethmoidal or directly transnasal; the latter may be done through one or both nostrils. The transeptal approach requires detaching all of the septum and using a nasal speculum; this technique is currently not used by the authors that have published papers about the endoscopic approach of the sellar region.

The transethmoidal approach requires removing the anterior and posterior ethmoid cells to reach the sphenoidal sinus and the sella turcica. It is also not used currently by those various medical groups. In the unilateral direct transnasal approach (done through one nostril), the endoscope and other surgical tools are inserted in the same nostril. In the bilateral direct transnasal approach, the endoscope is inserted in one nostril and the surgical tools are inserted in the other nostril.

Various authors contend that the unilateral approach is less invasive, as in this case the posterior nasal septum is not necessarily removed.^10^, ^12^, ^17^, ^18^, ^19^, ^20^ Those that defend the bilateral approach underline the possibility of conflict between the endoscope and other surgical tools due to lack of space when the approach is done through one nostril only.^21^, ^22^, ^23^, ^24^, ^26^

The bilateral transnasal endoscopic approach was used in all of the surgical procedures in this paper. One of the reasons for this choice is the increased comfort in using surgical tools in one nostril and the endoscope in the other. The decisive factor, however, was the simultaneous presence of the otorhinolaryngologists (responsible for handling the endoscope) and the neurosurgeon (responsible for handling the surgical tools) in the surgical field during the procedure, one on each side of the patient. This arrangement of the team would conflict if the unilateral approach were used.

The benefits of placing the team in this arrangement are significant. One of the criticisms of endoscopic surgery is that it is done with only one had, as the other is busy holding the endoscope. The surgeon, for instance, cannot aspirate the surgical field while removing the tumor. With the abovementioned arrangement, the otorhinolaryngologist holds the endoscope with one of his or her hands and a surgical instrument (generally the nasal aspirator) with the other. The neurosurgeon used one or both hands to manipulate the surgical tools through the other nasal fossa. This procedure requires integration between team members; with practice, however, it can be said that the hands operate as if belonging to the same person.

Van Lindert and Grotenhuis^24^ described a new tool by which it is possible to couple a malleable aspirator to an endoscope, allowing bimanual surgery. Although interesting, this option does not allow adequate free movement of the aspirator relative to the endoscope, which is important if there is significant bleeding.

Another commonly used possibility is to use endoscope holders. A mechanical device is used to fixate the endoscope (a mechanical arm or holder); after the sphenoidal sinus is opened, the endoscope is fixated, leaving the neurosurgeon with both hands free to perform the procedure.^7^, ^8^, ^12^, ^17^, ^18^, ^21^, ^22^, ^25^, ^27^, ^28^, ^29^, ^30^, ^31^, ^32^ Other authors prefer not to use an endoscope with a holder^14^, ^15^, ^19^, ^20^, ^24^, ^26^.

In the current study we did not use endoscope holders, as one of the most important advantages of the endoscope is its mobility; it can be rapidly repositioned without having to be released and refixated. The holding mechanism may not be sufficiently precise to hold the endoscope exactly in the desired position. Furthermore, endoscopes provide two-dimensional images with no depth, rather than the three-dimensional images offered by microscopes, which some authors consider a disadvantage.^12^, ^27^ One of the ways in which a notion of depth is obtained with an endoscope is to move it constantly backwards and forwards, using fixed anatomic landmarks as references.

A further disadvantage of using endoscopes, in our view, is that they may hinder the movement of the neurosurgeon’s surgical tools. Curettes and dissectors may be partially guided towards undesirable positions by the external contour of the endoscope; they may roll over the tip of the endoscope and result in abrupt movements.

Finally, at times it becomes necessary to clean the tip of the endoscope, which may become covered by condensation or blocked by blood; if the endoscope is fixed, time will be lost during the procedure. Some authors routinely used a cleaning system for the tip of the endoscope - irrigation with saline - to try to solve this problem.^7^, ^8^, ^12^, ^17^, ^27^, ^28^, ^29^, ^30^, ^31^, ^32^

Certain measures should be taken during the surgical approach to avoid complications. When the nasal fossae are prepared by placing cottonoid with vasoconstrictors between the middle turbinate and the nasal septum, the turbinate should be lateralized with great care so that it does not fracture close to the cranial base (lateral lamella of the lamina cribosa). This is the most fragile region of the anterior cranial base, at times with a thickness of 0.05mm, ten times less than the thickness of the ethmoidal roof.33 Septa within the sphenoidal sinus should not be routinely removed; these septa are frequently inserted close to the internal carotid artery or the optic nerve. If a better approach to the sellar region is required, these septa may be removed with great care with cutting forceps, which reduce the risk of causing a fracture line close to the internal carotid artery (with potentially disastrous consequences). Removal of these septa should only be attempted after carefully analyzing the images that reveal the relation between the septa and adjacent structures. Images should always be available in the operating room for consultation during surgery, as in functional endoscopic surgery of the paranasal sinuses.

The otorhinolaryngologist participates until the sphenoidal sinuses are opened when surgery is done using the sublabial, transeptal or transnasal approaches with an endoscope holder; the neurosurgeons takes on from there. In the approach that we used in this paper, the otorhinolaryngologist is responsible for providing the neurosurgeon with an adequate view of the surgical field until the end of the procedure. The neurosurgeon opens the floor of the sella and the dura-mater and removes the tumor with surgical tools placed in the left nostril, while the otorhinolaryngologist provides the advantages of the endoscopic view and a cleaner surgical field by placing the endoscope adequately and aspirating blood through the right nasal fossa.

It is important to explore the sellar region with an angulated endoscope after tumor removal to locate and remove tumor remains or to seek and correct sellar diaphragm injury, which could lead to cerebrospinal leaks. In the current approach the otorhinolaryngologist introduced a 45° endoscope into the sella turcica and rotated it by 360° to inspect the supra-sellar, para-selar, retro-selar and the sellar floor, while the neurosurgeon carefully moved the sellar diaphragm aside to remove tumor remains, if present.

Baussart et al.^34^ explored the sellar region with an angulated endoscope after removing tumors with a surgical microscope in 13 patients. The endoscope allowed them to find tumor remains that were not visible under microscopy in seven of these patients, all of which were resected. Contrary to other authors, Heilman et al.^14^ have stated that endoscopic inspection of the sella turcica following tumor removal does not bring significant benefit, as there is little room within the sella, and the tip of the endoscope repeatedly becomes clouded by blood. Here, we agree with Sonnenburg et al.^23^ who stated that “the participation of an otorhinolaryngologist throughout the surgical procedure is very important, and becomes essential when working with a neurosurgical team with significant experience in pituitary surgery but without formal training in the use of endoscopes and other paranasal sinus endoscopic surgical instruments.”

In our sample, 18 of 91 patients had already been operated by another surgical approach, five of them by craniotomy and 13 by the sublabial approach. Of the latter 13 patients, two had already been operated twice and one had undergone three procedures. The endoscopic transnasal approach is advantageous compared to other surgical approaches in cases of tumor recurrence, particularly in patients that were previously operated by the transeptal or sublabial approaches. The main advantage is not having to detach the nasal septum, which can be difficult in previously operated patients where the bone septum may have been removed and soft tissues are adhered to each other.

A further important point is that the sphenoidal sinus has been opened on other occasions, so that the first step in surgery takes places swiftly compared to the sublabial or transeptal approach, which in these cases is much more time consuming. Furthermore, endoscopes provide higher safety when progressing within the sphenoidal sinus in a condition of altered anatomy after previous surgery.^7^, ^36^

The mean hospital stay for patients that underwent the 95 procedures we studied was 5.4 days; in 81% of cases, patients remained in hospital for four days or less. The mean hospital stay of Sonnenburg et al.’s23 patients was 4.1 days (first 15-case groups), 4.5 days (second 15-case group) and 2.4 days (third 15-case group). The mean hospital stay in Cappabianca et al.’s28 series was 3.36 days in his first 100 cases operated by the transnasal endoscopic approach; the mean hospital stay had been 6.35 days for the last 100 patients operated sublabially. In the first 50 cases that were operated endoscopically, 24% of patients were discharged within two days of surgery.

In our series, 25.26% of patients were discharged two days after surgery. The higher mean hospital stay in our sample may in part be explained by the nature of our reference center; patients originated from various parts of the country and frequently remain in hospital for longer than necessary for recovery. Furthermore, our unit is part of a university teaching hospital for medical residents and students; rapid rotation of patients is not a significant priority. Another relevant point is the large number of macroadenomas in our series. Of 95 cases, 73 were cases of pituitary macroadenomas and four were cases of craniopharyngiomas. These tumors are frequently large and may be associated with neurological symptoms and complications that are not the aim of this study, but that may required a prolonged hospital stay.

The endoscopic transnasal approach was feasible in all of the sample patients regardless of age, and the etiology and characteristics of tumors. Shikani and Kelly16 described a case in which they attempted to biopsy and remove the tumor by the endoscopic transnasal approach but were unable to find the ostium of the spnenoidal sinus; they then opted for the endoscopic transethmoidal approach.

Regarding the technical difficulties (need for removing the middle turbinate and septal deviation and the need for image intensifiers), we should bear in mind that the middle turbinate is an important structure for nasal and sinus function. It helps direct the airflow within the nasal fossae and is part of the so-called ostial-meatal complex, a functional unit responsible for anterior paranasal sinus ventilation and drainage.50 Removal of this structure reduces the surface area of the nasal mucosa, reducing its capability to warm and humidify inhaled air and transporting nasal secretions.

Many authors have reported partial or total removal of the middle turbinate to facilitate the approach to sphenoidal sinus.^10^, ^11^, ^15^, ^19^, ^21^ There was no need to remove the middle turbinate in any of our cases, which is aligned with the intention of other groups that always seek to preserve the middle turbinate whenever possible.^16^, ^25^, ^27^, ^30^, ^33^, ^36^, ^38^ Topical use of cottonoids with adrenalin at 1:1000 may have helped to locate the ostium of the sphenoidal sinus.51 Intense vasoconstriction and decreased mucosal edema resulting from the use of adrenalin facilitate the approach to the sphenoethmoidal recess without the need to remove the middle turbinate partially or totally.

There was no need to remove septal deviations to facilitate the approach to the sphenoidal sinus in any of the cases. The endoscopic transnasal approach was possible even in patients with marked nasal septum deviations; the endoscope and surgical tools were maneuvered over or below the deviation. Most authors do not comment on the need for nasal septum deviations during the endoscopic transnasal approach. Stamm et al.^15^ have reported doing septoplasty, if needed; Moreland et al.^18^ are the only authors to state that they found no need to correct septal deviation for approaching the sellar region in three cases.

The last difficulty found in the surgical approach we studied was the need for image intensifiers to confirm the trajectory of surgical tools. It is used routinely in sellar region surgery with microscopes and by some authors that use the endoscopic approach.^12^, ^20^

We required image intensifiers to confirm the trajectory of surgical tools in two cases, both of which had conchal type sphenoidal sinuses. This finding is similar to those of Cappabianca et al.,^42^ who only use this recourse in cases of pre-sellar or conchal sphenoidal sinuses. The conchal type sinus does not reach the body of the sphenoid bone and is separated from the sella turcica by a thick bony wall. In this situation it is not possible to locate anatomical landmarks such as the sphenoidal plane, the clivus and the bony prominences of the internal carotid arteries, thus the need for image intensifiers. Other authors have underlined the usefulness of navigation systems to help locate anatomical landmarks, particularly in cases of tumor recurrence.^39^, ^40^

Two important surgical intercurrences were noted in this paper, perioperative bleeding and sellar diaphragm injuries. Most of the severe complications of nasosinusal endoscopic surgery (perforation of the ethmoidal roof, optic nerve injury) occur in those cases operated under unfavorable visibility conditions due to intense bleeding.^41^

The anesthesiologist has a fundamental role in controlling perioperative bleeding. Rigorous control of blood pressure levels and the use of endovenous, rather than inhaled anesthetics, allow a surgical approach with minimal bleeding. Topical vasoconstrictor use also has a significant effect for controlling hemorrhage.^38^ Kassam et al.^42^ described a variety of techniques for controlling perioperative bleeding in sellar region endoscopic surgery, and brought attention to the use of hemostatic substances that promote platelet adhesion and clotting. We used one of these products (Spongostan powder®, Johnson & Johnson) in those cases where undesirable sellar region or nasal mucosa bleeding was present in our patients.

There were no cases of interruption of surgery due to perioperative nasal bleeding in our sample or cases that required blood transfusion. This finding differs from those reported by Cappabianca et al.;^8^ they reported one case of bleeding of the internal carotid artery during surgery. Nasal tamponade was not used after surgery in our sample, which is different from Nasseri et al.’s43 study of 180 patients operated by the endoscopic approach; they reported having used nasal tamponade after surgery in 17 cases due to “concerns with nasal bleeding.” Surgical reconstruction - filling in the sella with fat and biological glue (Beriplast®) - was required for sellar diaphragm injury during tumor removal in 13 cases (13.68%); in two of these cases (3.15%) external lumbar drainage was done additionally. None of these cases developed cerebrospinal leaks or meningitis postoperatively. Some authors do not specify the number of patients in which the sellar diaphragm was injured, believing that this is a minor intercurrence; they merely explain the corrective measures.^11^, ^12^, ^19^, ^21^, ^27^, ^30^

Findings vary among the authors that report the number of perioperative cerebrospinal leaks. Our percentage (13.68%) is close to that reported by White et al.^22^ (12%) Cappabianca et al.^44^ (14.11%) and Sonnenburg et al.^23^ (15.55%). Other authors have reported higher rates compared to our series.^10^, ^14^, ^20^, ^36^, ^43^ Stamm et al.^15^ reported that 6.4% of their patients required perioperative correction of sellar diaphragm injuries.

Complications due to the endoscopic approach in our sample were as follows: postoperative bleeding, postoperative cerebrospinal leak and meningitis. Otorhinolaryngological care was required to correct postoperative bleeding in 8.42% of patients. Of these, one was treated by lavage of the nasal cavity with saline. Gelfoam® placed in the nasal fossae for 24 hours was used to control bleeding in another patient. Anterior nasal tamponade was used in three other cases, two of them for 24 hours and one for 48 hours. Two patients required nasal anterior and posterior tamponade, one for 24 hours and one for 72 hours. Finally, one patient required surgical cauterization of the nasal mucosa. Aust et al.^45^ reported a postoperative bleeding rate of 14.28%; many other authors have reported lower rates compared to our series in this paper.^8^, ^15^, ^22^, ^23^, ^27^, ^32^

The criteria used to define an episode of nasal bleeding may be an explanation of this finding. Nasal bleeding was defined as present whenever an otorhinolaryngological assessment was required postoperative to evaluate bleeding. Endocrinology medical residents made this request and otorhinolaryngology medical residents made the evaluation itself, at times during weekends or at night. After the initial treatment, the team that had operated the case was called upon, if needed.

Five of eight cases of nasal bleeding were solved by simple procedures, and might have been managed without the need for tamponade. Two patients required anterior and posterior tamponade; in one of these cases, tamponade was ceased 24 hours later after the surgical team assessed the case, with no undesirable consequences. The patient that underwent cauterization under general anesthesia had diffuse bleeding of the nasal mucosa nasal, and had been taking Ginkgo-biloba. The possibility that bleeding in this case might have occurred due to the anticoagulant effect of this plant extract cannot be discarded, or that surgical treatment was necessary to stop the bleeding. Hemorrhage due to Ginkgo-biloba in various anatomical sites has been reported in the; the American Anesthesiology Society currently recommends that Ginkgo-biloba therapy be interrupted two weeks before any surgery.46 Bent et al.^47^ did a systematic review of the literature about Ginkgo-biloba and bleeding, and reached the conclusion that there might be a cause-effect relation between both.

Cerebrospinal leaks occurred in eight cases (8.42%) and meningitis occurred in two cases (2.11%). Six of the eight leaks were detected on the first week postoperatively; the remaining two cases were detected on the third week after surgery. The two latter cases that presented rhinoliquorrhea developed meningitis.

A few authors have reported higher rates of postoperative cerebrospinal leaks compared to our study. Aust et al.^45^ reported a 14.28% rate of postoperative cerebrospinal leaks. White et al.^22^ reported a 12% rate of postoperative cerebrospinal leaks and a 2% rate of meningitis. Most authors, however, have reported lower rates of this complication compared to our series. Sethi and Pillay^11^ and Rudnik et al.^36^ reported a 5% rate of postoperative cerebrospinal leaks. Sonnenburg et al.^23^ reported a 4.44% rate of postoperative cerebrospinal leaks a 2.22% rate of meningitis. Nasseri et al.^43^ reported a 4.4% rate of postoperative cerebrospinal leaks, and Jho and Carrau^12^ reported a 3.8% rate of postoperative cerebrospinal leaks. Cappabianca et al.^44^ reported a 2.3% rate of this complication in their series.

The fact that 8.42% of the patients in our series had cerebrospinal leaks may be due to the surgical learning curve of the team. Most of the groups that use endoscopic surgery already had vast experience in microscopic pituitary surgery before changing over to the endoscopic technique; in our case the team experience was gained mostly in endoscopic surgery. A study by Ciric et al.^48^ reveals a significant reduction in morbidity in microscopic transsphenoidal pituitary surgery after 200 and even 500 operated cases; our sample assessed the first 95 such procedures done by our team.

A further factor that may have increased the occurrence of postoperative cerebrospinal leaks is the nature of our series, as mentioned above. Of 95 procedures, 73 were for the treatment of pituitary macroadenomas and four were craniopharyngiomas. Six of the eight patients that developed postoperative cerebrospinal leaks had macroadenomas that extended outwards from the sella (supra-sellar and para-sellar); one case was a craniopharyngioma that extended supra-sellarly and the other had a Ratke’s pouch cyst that extended to the supra-sellar region. Otorhinolaryngologists have an important role in the correction of postoperative cerebrospinal leaks. The endonasal endoscopic approach is currently the preferred route for the treatment of these cases. Silva et al.49 reported success rates over 90% for the endoscopic surgical treatment of postoperative cerebrospinal leaks after a first surgery.

Advantages of the endoscopic approach to the sellar region include: increased vision of the surgical field, decreased postoperative discomfort, greater respect for nasal structures and reduced hospital stay. The disadvantages are: the need for a learning curve, the need to develop specific abilities to handle endoscopes and surgical tools, the lack of three-dimensional vision and the need for carefully controlling perioperative bleeding.^27^ Bleeding from branches of the sphenopalatine artery was the only complication in our sample, among those listed for the sublabial surgical approach (anesthesia of the upper lip and teeth, saddle nose, perforation of the nasal septum, anosmia, maxillary diastasis or fracture of the hard palate, fracture of the orbit, fracture of the lamina cribosa and bleeding of branches of the sphenopalatine artery). The explanation may be that in this approach no instrument passes through the mouth, effectively beginning when the ostium of the sphenoidal sinus is opened.^8^

The history of endoscopes is an example of how technological developments have an influence on medicine. Philipp Bozzini introduced endoscopes in medicine not more than 200 years ago, and today this device is part of the daily practice of various specialties. Its use in otorhinolaryngology brought undeniable improvements for diagnosis and for reducing the morbidity of a variety of surgical procedures. Its use is very promising in surgery of the anterior cranial base, although it still needs to pass the test of time. The experience attained in various specialties should be gathered together in this context for the benefit of patients. As Harvey Cushing stated in the beginnings of transphenoidal surgery in 1912: “Performance is progressively simplified by the combined suggestions and experience of many.”^50^

## CONCLUSIONS

1. The transnasal endoscopic approach to sellar tumors may be done with minimally invasive surgery preserving nasal structures in the 95 procedures that we investigated, regardless of the patient’s age, and the tumor nature and etiology.
2. The main intercurrence during surgery was injury of the sellar diaphragm, seen in 13 procedures (13.68%). All of these cases were managed perioperatively, with no postoperative complications.
3. The main postoperative complications were nasal bleeding (8.42%), cerebrospinal leaks (8.42%) and meningitis (2.11%).
